# Longitudinal trends and interelations of mitochondrial function in adults and pediatric critically ill patients

**DOI:** 10.1186/2197-425X-3-S1-A287

**Published:** 2015-10-01

**Authors:** AM Spanaki, T Tavladaki, H Dimitriou, AV Kozlov, JC Duvigneau, S Dumitrescu, E Kondili, D Georgopoulos, G Briassoulis

**Affiliations:** PICU, University of Crete/University Hospital, Heraklion, Greece; Paediatric Haematology Oncology, University of Crete, Medical School, Heraklion, Greece; Ludwig Boltzmann Institute for Experimental and Clinical Traumatology in the AUVA, Vienna, Austria; University of Veterinary Medicine, Vienna, Austria; ICU, University of Crete/University Hospital, Heraklion, Greece

## Introduction

Bioenergetic failure due to mitochondrial dysfunction has been implicated as an important pathophysiological mechanism underlying poor outcome in critical illness.

## Objectives

We examined the longitudinal changes of ATP, N02¯ and N03¯ in patients with severe sepsis (SS) or trauma-related systemic inflammatory response syndrome (SIRS) compared to healthy-controls (H) and their relations to intracellular heat shock proteins (HSP)-72 and -90α, metabolism and outcome.

## Methods

Seventy-eight adults (SS/22; non-infectious SIRS /23; healthy (H)/33) and sixty-two children (SS/15; non-infectious SIRS /20; healthy (H)/27) were studied. Blood samples obtained on days 1, 3 and 5. Energy expenditure (EE) of patients was measured with the Gas Module E-COVX. HSPs expressions in monocytes (m) or neutrophils (n) were determined using flow cytometry. ATP concentrations were measured by the luciferase luminescent assay. N02¯ and N03¯ determination was performed using the Sievers Nitric Oxide Analyzer.

## Results

In both, adults and children, mitochondrial bioenergetics showed different longitudinal trends for survivors and non-survivors. The nitrite/nitrate ratio increased longitudinally in critical illness (p < 0.05). Among adult survivors, N03¯ (26422 ± 19368 vs. 13807 ± 3740nM, p < 0.04) and ATP concentrations (503 ± 645 vs. 185 ± 192nM) decreased from day 1 to 3, and lactate from days 1 to 3 to 5 (15 ± 11 vs. 10 ± 4 vs. 8 ± 5mMol/L, p < 0.001). Survivors had higher ATP, N03¯ (p < 0.04), N02¯ (p < 0.04), V02 (p < 0.0001), VC02 (p < 0.0001), EE (p < 0.04) on day 1, N02¯ (p < 0.05) on day 3, and lower lactate levels on days 1 and 5 (p < 0.03), compared to non-survivors. Non-survived children had lower SID (28 ± 5 vs. 34 ± 4, p < 0.03) and mHSP72 (16 ± 13 vs. 23 ± 15 MFI, p < 0.05) on days 1, 5.

## Conclusions

Our data implicate bioenergetic failure and a drop in NO synthesis in white blood cells as possible pathophysiological mechanisms contributing to mortality. Our data suggest that in critically ill patients NO metabolism is linked to HSP72 and has a protective impact on mitochondrial function.

## Grant Acknowledgment

This research has been co-financed by the European Union (European Social Fund (ESF)) and Greek national funds through the Operational Program "Education and Lifelong Learning" of the National Strategic Reference Framework (NSRF)-Research Funding Program: THALES.
